# Transmitter-Free Interfacial Sensitization in ZnSe/ZnS Quantum Dots Enables Efficient Upconversion

**DOI:** 10.3390/nano16140843

**Published:** 2026-07-09

**Authors:** Yan Wang, Weizhe Hu, Haicheng Liu, Linhai Fu, Ting Yang, Xian Lin, Ye Dai, Guohong Ma, Quan Zhang, Jie Zhang

**Affiliations:** 1 Department of Physics, Shanghai University, Shanghai 200444, China; wangyan_@shu.edu.cn (Y.W.); liuhaicheng@shu.edu.cn (H.L.); fulinhai@shu.edu.cn (L.F.); yangting@shu.edu.cn (T.Y.); linxian01@shu.edu.cn (X.L.); yedai@shu.edu.cn (Y.D.); ghma@staff.shu.edu.cn (G.M.); 2 Institute for Quantum Science and Technology, Shanghai University, Shanghai 200444, China; 3 Institute for Chemical Research, Kyoto University, Kyoto 611-0011, Japan; hu.weizhe.83a@st.kyoto-u.ac.jp; 4 State Key Laboratory of Advanced Fiber Materials, College of Materials Science and Engineering, Donghua University, Shanghai 201620, China; 5 Shanghai Engineering Research Center for Integrated Circuits and Advanced Display Materials, Shanghai University, Shanghai 200444, China

**Keywords:** quantum dots, photon upconversion, triplet–triplet annihilation, triplet energy transfer, transmitter ligands

## Abstract

Triplet–triplet annihilation upconversion (TTA-UC) from the visible to ultraviolet (UV) holds promise for UV-driven photochemistry, yet the design principles governing interfacial triplet energy transfer (TET) and overall efficiency remain debated. Here, we compare two sensitization strategies for TTA-UC using ZnSe/ZnS core/shell quantum dots, including transmitter-mediated (with 4-biphenylcarboxylic acid, BCA) and transmitter-free direct assembly. Two high-triplet-energy annihilators, 2,6-di-tert-butylnaphthalene (DTBN) and p-terphenyl (TP), are investigated. Time-resolved photoluminescence reveals that while DTBN exhibits faster interfacial TET (*k*_TET_ = 0.030 ns^−1^ vs. 0.022 ns^−1^ for TP in direct assemblies), its low fluorescence quantum yield (4.77% vs. 54.59% for TP) severely limits UC efficiency. Consequently, the direct ZnSe/ZnS-TP assembly achieves a maximum UC efficiency of 7.9%, outperforming both its BCA-mediated counterpart (6.9%) and DTBN-based systems (≤1.5%). These findings establish that simplifying the donor–acceptor interface while prioritizing the annihilator emissivity can yield superior performance, providing a key design principle for high-efficiency visible-to-UV upconversion materials.

## 1. Introduction

Photon upconversion based on triplet–triplet annihilation (TTA-UC) has emerged as a versatile strategy for converting low-energy photons into higher-energy emission under weak incoherent light, offering promising applications in photocatalysis, solar energy conversion, and bioimaging [1,2,3,4,5,6,7]. In particular, visible-to-ultraviolet (vis-to-UV) TTA-UC holds significant potential for enabling UV-driven processes such as photopolymerization, environmental remediation, and organic synthesis, where direct UV sources are limited or hazardous [8,9,10,11,12,13]. Compared with conventional molecular triplet sensitizers such as heavy-metal porphyrins (e.g., Pt/Pd complexes), semiconductor quantum dots (QDs) offer distinct advantages, including broad tunable absorption and large extinction coefficients, which can be harnessed for efficient triplet energy transfer (TET) [14,15,16,17,18,19,20,21,22,23,24,25,26,27,28,29]. Importantly, the energy loss induced by singlet–triplet energy gaps (ΔE_ST_) can be reduced by the negligible singlet–triplet splitting (ΔE_ST_ < 15 meV) of QDs [30,31,32].

A key challenge in QD-sensitized TTA-UC lies in the interfacial energy transfer from the QD triplet state to the molecular annihilator. To facilitate this process, “transmitter ligands”, often aromatic carboxylates such as biphenylcarboxylic acid (BCA), have been widely adopted to mediate triplet transfer from QDs to annihilators [33,34,35]. While effective, this relay approach introduces additional complexity, potential energy loss channels, and constraints on annihilator selection, thereby complicating material design [36,37,38,39,40,41,42,43,44,45]. Recent reports have demonstrated transmitter-free direct QD–annihilator coupling in specific systems, such as ZnSe/ZnS QDs with 2,5-diphenyloxazole, where trap-enabled long exciton lifetimes facilitate efficient TET without the need for intermediate transmitter ligands [46]. This approach simplifies the interfacial energy transfer pathway, offering a promising strategy for improving the overall upconversion efficiency. Nevertheless, further improving the UC efficiency in such transmitter-free systems remains challenging, primarily due to limitations in the interfacial energy transfer kinetics and annihilator photophysical properties.

In this study, we construct a ZnSe/ZnS core–shell QD-based TTA-UC platform aimed at vis-to-UV conversion. We employ two high-triplet-energy annihilators, *p*-terphenyl (TP) and 2,6-di-tert-butylnaphthalene (DTBN), both possessing sufficiently high singlet energies to emit in the UV region upon triplet–triplet annihilation. Through a systematic comparison of BCA-mediated relay sensitization and direct QD-annihilator assemblies, we investigate the interplay between interfacial energy transfer kinetics, annihilator photophysical properties, and overall upconversion efficiency. Our work not only demonstrates that direct sensitization can match or exceed relay-based systems in efficiency but also provides fundamental insights into the design principles for high-performance, low-toxicity upconversion materials operating in the vis-to-UV spectral range.

## 2. Materials and Methods

### 2.1. Materials

The zinc acetate (Zn(OAc)_2_, 99.5%), 1-octadecene (ODE, 90%), selenium (Se, 99.99%), 4-biphenylcarboxylic acid (BCA, 99%), 2,6-di-tert-butylnaphthalene (DTBN, 98%), *p*-terphenyl (TP, 99.9%), perylene (98%) and hexane were purchased from Shanghai Titan Scientific Co., Ltd. (Shanghai, China). Acetone and sublimated sulfur (S, 99.5%) were purchased from Sinopharm Chemical Reagent Co., Ltd. (Shanghai, China). Oleic acid (OA, >85%) was purchased from Tokyo Chemical Industry Co., Ltd. (Tokyo, Japan). All chemicals were used as received without further purification.

### 2.2. Synthesis of ZnSe/ZnS Core–Shell Quantum Dots

ZnSe/ZnS core/shell quantum dots (QDs) were synthesized by a hot-injection method [34]. Selenium (Se, 0.8 mmol) and sulfur (S, 0.5 mmol) precursors were prepared by dissolving their powders in ODE (6 and 5 mL, respectively). A zinc shell precursor was prepared by mixing Zn(OAc)_2_ (2.0 mmol) with OA (2 mL) and ODE (5 mL). For synthesis, Zn(OAc)_2_ (0.8 mmol, 146.8 mg), OA (0.8 mL), and ODE (10 mL) were degassed at 120 °C for 60 min, then heated to 300 °C under N_2_ for 60 min to form Zn–oleate. After adjusting to 290 °C, 3 mL of Se precursor was rapidly injected, and the reaction was maintained for 8 min to grow ZnSe cores (core size tunable by Se injection amount). The ZnS shell was grown by three successive additions at 8 min intervals (0.35 mL Zn precursor, 0.50 mL S precursor, and 0.35 mL Zn precursor), followed by an additional 5 min at 290 °C and cooling to room temperature. QDs were purified twice by hexane/ethanol precipitation and centrifugation and finally redispersed in hexane.

### 2.3. Preparation of ZnSe/ZnS-BCA–Annihilator and ZnSe/ZnS–Annihilator Composites

ZnSe/ZnS-BCA complexes were obtained by adding BCA to ZnSe/ZnS QDs in hexane and stirring for 20 min at room temperature. BCA-mediated and transmitter-free samples were prepared by mixing the corresponding QD (or QD-BCA) dispersions with TP or DTBN stock solutions, diluting with hexane to a final annihilator concentration, and adjusting the quantum dot optical density (OD) to 0.1. Samples were loaded into sealable 1 cm quartz cuvettes equipped with septum caps. For nitrogen-atmosphere measurements, high-purity N_2_ was bubbled through the sample solutions for 20 min to remove dissolved oxygen, and the cuvettes were then sealed immediately before optical measurements. For comparison, selected samples were also measured under ambient atmosphere without nitrogen purging.

### 2.4. Characterization

UV–vis absorption spectra were recorded on a Shimadzu UV-3600 UV–Vis–NIR spectrophotometer (Shimadzu Corporation, Tokyo, Japan). Steady-state photoluminescence (PL) spectra, photoluminescence excitation (PLE) spectra, photoluminescence quantum yields (PLQYs), and time-resolved PL (TRPL) decay curves were measured using an FLS1000 spectrometer (Edinburgh Instruments Ltd., Livingston, UK) equipped with a single-photon counting detector. The steady-state PL, PLE, and PLQY measurements were performed using a xenon lamp (SOFN Instruments Co., Ltd., Beijing, China) as the excitation source, with the excitation or emission wavelength selected according to the specific sample and measurement purpose. TRPL decay curves were measured under 375 nm pulsed excitation by monitoring the QD band-edge emission at the corresponding emission maximum, typically in the range of 410–420 nm. TEM images were acquired on a JEM-2100F transmission electron microscope (JEOL Ltd., Tokyo, Japan).

TTA-UC emission spectra were measured under 390 nm excitation generated by frequency doubling the 780 nm output of a Spectra-Physics Spitfire Ti:sapphire femtosecond regenerative amplifier system using a BBO crystal. The laser operated at 1 kHz with a pulse duration of approximately 120 fs. The excitation power density was calculated from the measured average optical power and the illuminated area at the sample position. Detailed information on the optical setup and power density determination is provided in Section S4 of the Supplementary Materials.

## 3. Results and Discussion

**Figure 1:**
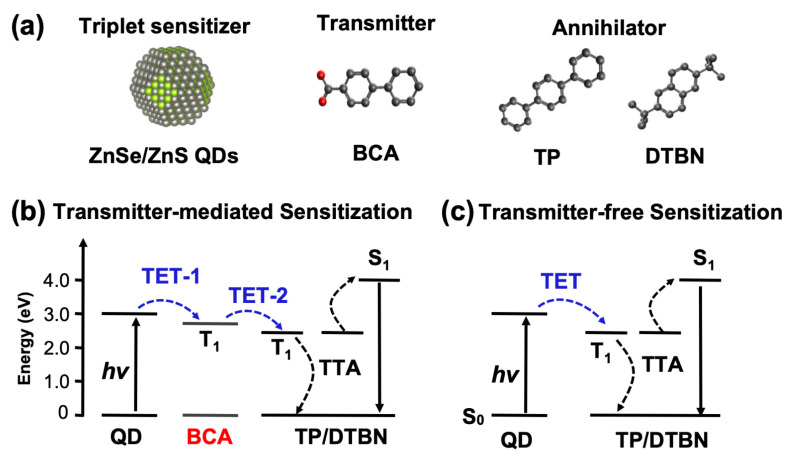
(**a**) Structures of the triplet sensitizer (ZnSe/ZnS QDs), transmitter (BCA), and annihilators (TP, DTBN). Schematic diagrams of (**b**) BCA-mediated relay and (**c**) transmitter-free direct sensitization strategies for TTA-UC.

The structures of the QD–molecule composite are illustrated in Figure 1a. ZnSe/ZnS core/shell QDs were selected as triplet sensitizers due to their low toxicity and ability to absorb visible light. BCA was employed as triplet transmitter, given its suitable triplet energy level for mediating energy transfer from QDs to annihilators. DTBN and TP, with high triplet energies, were chosen as UV-emitting annihilators. DTBN has been previously employed as a UV-emitting annihilator in TTA-UC systems [34]. The BCA-mediated relay and transmitter-free direct sensitization strategies are schematically illustrated in Figure 1b and Figure 1c, respectively.

**Figure 2:**
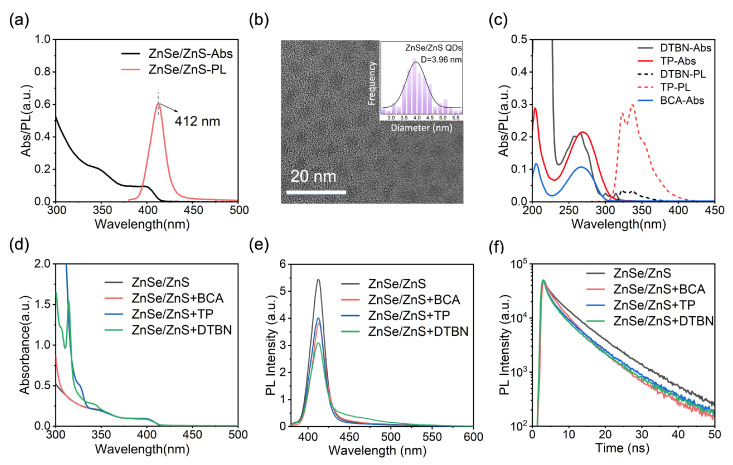
(**a**) Absorption and PL spectra of ZnSe/ZnS QDs. (**b**) TEM image of ZnSe/ZnS QDs; the inset shows the size distribution histogram. (**c**) Absorption and PL spectra of TP and DTBN and absorption spectrum of BCA. Absorption spectra (**d**), steady-state PL spectra (**e**), and time-resolved PL decays (**f**) of ZnSe/ZnS QDs with and without BCA functionalization compared with direct composites of TP or DTBN. The steady-state PL spectra were measured under xenon lamp excitation at 375 nm for (**a**,**e**), and at 260 nm for (**c**).

ZnSe/ZnS core–shell QDs were synthesized via hot-injection and multistep shell growth, exhibiting a well-resolved first excitonic absorption at ~400 nm and narrow band-edge PL peak at 412 nm (full width at half-maximum = 18 nm; Figure 2a). The PLQY of the ZnSe/ZnS QDs was measured to be 35.19% (Figure S1a). Transmission electron microscopy (TEM) reveals monodisperse spheres with an average diameter of 3.96 nm (Figure 2b). The absorption spectra of BCA, DTBN, and TP, together with the PL spectra of DTBN and TP, are shown in Figure 2c. Both annihilators exhibit intense π-π* absorption bands below 320 nm and structured UV fluorescence between 300–400 nm. Critically, their fluorescence quantum yields differ by an order of magnitude (Φ_PL_ = 54.59% for TP versus 4.77% for DTBN; Figure S1b,c). Density functional theory calculations (CAM-B3LYP/6–311g (2d, p)) yield singlet (S_1_) and triplet (T_1_) energies of 4.08 eV and 2.63 eV for TP, 4.08 eV and 2.45 eV for DTBN, and 4.31 eV and 2.79 eV for BCA, respectively (Table S1). The T_1_ energy of BCA lies below the QD band energy (~3.0 eV estimated from PL results) and above those of TP and DTBN, supporting the energetic feasibility of stepwise TET from QDs to BCA and subsequently to the annihilators. In addition, both annihilators possess T_1_ energies sufficiently below the QD band energy to enable exergonic direct TET.

To probe the interfacial TET, we first functionalized ZnSe/ZnS QDs with BCA (0.5 mM) as a molecular transmitter (Figure 2d). BCA coordination quenches the steady-state QD PL (Figure 2e) and accelerates the PL decay in time-resolved measurements (Figure 2f). The TRPL decay curves were measured under 375 nm pulsed excitation by monitoring the QD band-edge emission at the corresponding emission maximum. Thus, the shortened QD PL lifetime indicates an additional interfacial exciton-deactivation pathway, consistent with efficient TET from QDs to surface-anchored BCA. Biexponential fitting yields a TET rate *k*_TET_ = 0.040 ns^−1^ and efficiency Φ_TET_ = 22.5% (Table 1). Upon addition of annihilators (3 mM) to QD-BCA dispersions, further PL quenching is observed (Figure S2), indicating additional TET pathways from QD to free-diffusing annihilators. Notably, the apparent TET metrics derived from QD PL quenching suggest stronger interfacial extraction for DTBN-containing composites, with *k*_TET_ = 0.065 ns^−1^ and Φ_TET_ = 32.1% for QD-BCA-DTBN compared with 0.035 ns^−1^ and 20.2% for QD-BCA-TP (Table 1). The shortened QD PL lifetimes indicate additional interfacial exciton-deactivation pathways after coupling with the annihilator molecules. Based on the lifetime variations, *k*_TET_ and Φ_TET_ were estimated to compare the TET between different QD–molecule systems, rather than to directly measure molecular triplet formation or triplet-state population.

In transmitter-free direct composites, steady-state PL quenching was observed upon mixing QDs with annihilators, with the residual PL intensity decreasing in the order QDs > QD-TP > QD-DTBN (Figure 2e). Time-resolved PL (Figure 2f) reveals accelerated decays for both directly coupled systems, with faster extraction for DTBN (*k*_TET_ = 0.030 ns^−1^, Φ_TET_ = 17.9%) than for TP (*k*_TET_ = 0.022 ns^−1^, Φ_TET_ = 13.8%; Table 1). These results establish a consistent pattern that DTBN exhibits faster interfacial TET than TP in both mediated and direct architectures, suggesting more efficient triplet harvesting from the QDs. This observation might be attributed to the low triplet energy of DTBN (2.45 eV vs. 2.63 eV for TP), which provides a larger driving force for TET from the QD band edge (~3.0 eV). However, as will become evident in the following sections, faster TET does not necessarily translate to superior upconversion performance.

To complement the lifetime-based analysis and further examine the interfacial quenching behavior, atmosphere-dependent steady-state PL measurements were performed under ambient air and N_2_ atmosphere. As shown in Figure S3, the QD band-edge emission is markedly quenched in all QD–annihilator composite samples under both atmospheres. The PL quenching efficiency was estimated from the integrated QD PL emission area according to ($\eta_{q}=1-I_{DA}/I_{D}$), where *I*_D_ and *I*_DA_ denote the integrated QD PL intensities in the absence and presence of annihilator molecules, respectively. The calculated values are indicated in Figure S3. The observed PL quenching confirms that additional interfacial exciton-deactivation channels are introduced upon coupling the QDs with annihilator molecules. Moreover, the stronger quenching observed in the DTBN-containing and BCA-mediated systems is broadly consistent with the TRPL-derived TET trend. It should be noted that these steady-state PL quenching efficiencies are used as complementary evidence for comparing interfacial quenching behavior, rather than as direct quantitative measurements of TET efficiency.

**Figure 3:**
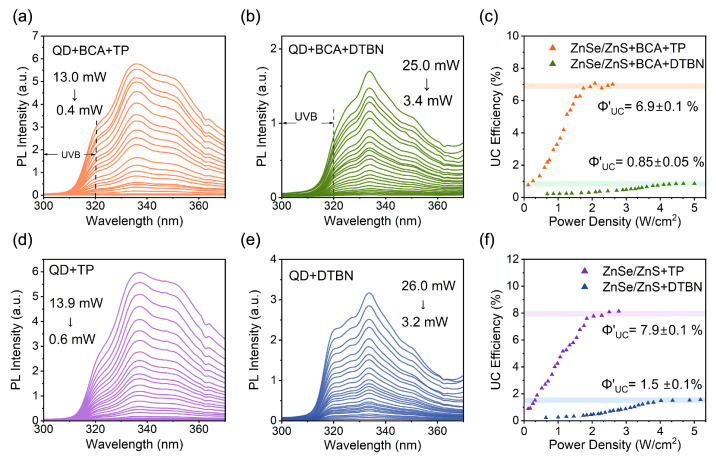
Power-dependent UC emission spectra of QD-BCA-TP (**a**), QD-BCA-DTBN (**b**), QD-TP (**d**) and QD-DTBN (**e**) under 390 nm excitation. Corresponding UC efficiencies versus excitation power density for BCA-functionalized (**c**) and direct assemblies (**f**). The emission range of 310–370 nm corresponds to 3.35–4.00 eV.

Power-dependent UC emission spectra were measured under 390 nm excitation (3.18 eV). Both direct and mediated assemblies produce characteristic UV emission matching the S_1_ → S_0_ fluorescence of the corresponding annihilator (310–370 nm; Figure 3a,b,d,e). Control experiments showed no detectable upconversion emission from QD-only, TP-only, or DTBN-only samples (Figure S4). To further support the assignment of the relevant emission bands, conventional photoluminescence excitation (PLE) spectra were measured (Figures S5 and S6). The PLE spectra monitored at 420 nm indicate that the emission around 420 nm is mainly associated with ZnSe/ZnS QD-related excitonic emission, while the differences observed for the QD–annihilator composites suggest the possible influence of molecular absorption and interfacial interactions. In contrast, the PLE spectra of TP and DTBN monitored at 335 nm are distinct from those of the QDs and support the assignment of the ultraviolet emission bands to annihilator fluorescence.

The integrated UC intensity exhibits the canonical TTA-UC behavior, showing a quadratic dependence at low power densities and transitioning to linear scaling above a threshold power density (Ith) (Figure S7). Ith values are consistently lower for TP-based systems (1.5–1.6 W cm^−2^) than for DTBN-based systems (3.9–4.2 W cm^−2^), indicating more efficient triplet accumulation in TP assemblies. The upconversion efficiency (Φ′_UC_, normalized to 100%) was determined as a function of excitation power (Figure 3c,f; Table 1), and the detailed calculation method, including the equation and relevant parameters, is provided in Section S4 of the Supplementary Materials. Strikingly, despite DTBN’s faster interfacial TET, its UC efficiency remains drastically lower across all architectures. In direct composites, QD-TP achieves a maximum Φ′_UC_ of 7.9%, compared with only 1.5% for QD-DTBN. Similarly, in mediated composites, QD-BCA-TP reaches 6.9% versus 0.85% for QD-BCA-DTBN. This difference suggests that despite slower initial TET, subsequent processes, including TTA and PL in TP, may possess higher efficiency.

The oxygen sensitivity of the UC emission was further examined by comparing the spectra under ambient air and N_2_ atmosphere. As shown in Figure S8, the UC emission is markedly enhanced after nitrogen purging but strongly suppressed under ambient air, which is consistent with a triplet-mediated TTA-UC mechanism because molecular triplet states involved in TTA are readily quenched by dissolved oxygen.

To further probe the roles of annihilator loading and transmitter coverage, we varied the concentrations of TP/DTBN and BCA in the corresponding composites. For direct composites, the TP-based system showed a strong concentration dependence, with the highest Φ′_UC_ obtained at 3 mM TP, whereas lower TP concentrations led to substantially reduced efficiencies (Figure S9). In contrast, DTBN-based direct composites showed only limited improvement with concentration, remaining far less efficient than the TP analogue (Figure S9). For BCA-mediated systems, increasing the BCA concentration caused a monotonic decrease in Φ′_UC_ for both TP and DTBN, indicating that excessive transmitter loading is detrimental to overall upconversion (Figures S10 and S11).

This stark contrast directly implicates annihilator photophysics as the dominant bottleneck. The UC efficiency in direct assemblies can be expressed as Φ′_UC_ = Φ_TET_·Φ_TTA_·Φ_PL_, where Φ_TTA_ is the TTA efficiency, which approaches a spin-statistical limit at high powers. Under our operating conditions (above Ith), Φ_TTA_ is comparable across systems, reducing the governing factors to Φ_TET_ and Φ_PL_. Although DTBN exhibits higher Φ_TET_, its 10-fold lower Φ_PL_ (4.77% vs. 54.59%) overwhelms this advantage, resulting in inferior UC performance.

**Figure 4:**
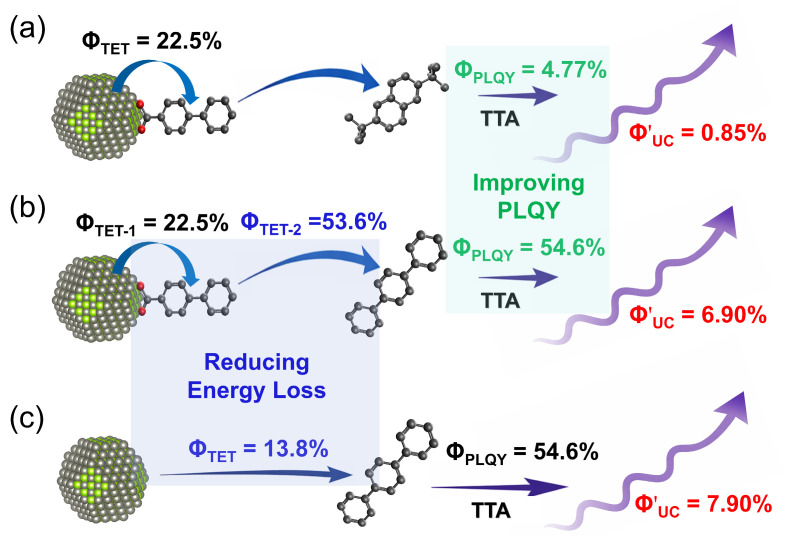
Schematic illustration of energy transfer pathways in (**a**,**b**) BCA-mediated and (**c**) direct ZnSe/ZnS-annihilator assemblies, highlighting the trade-off between interfacial transfer kinetics and annihilator emissivity.

The superior performance of the direct ZnSe/ZnS-TP assembly (Φ′_UC_ = 7.9%) over its BCA-mediated counterpart (6.9%) demonstrates that simplifying the donor–acceptor interface can enhance the overall efficiency in this system, even when the initial TET rate is slower. This seemingly counterintuitive result highlights that cumulative energy losses, potentially through non-radiative decay within the transmitter or inefficient relay to the annihilator, can offset gains in the initial harvesting step. In contrast, direct coupling minimizes such losses, allowing the annihilator’s intrinsic emissivity to dominate, as illustrated in Figure 4.

For transmitter-mediated assemblies, the overall upconversion efficiency follows the cascade Φ′_UC_ = Φ_TET-1_·Φ_TET-2_·Φ_TTA_·Φ_PL_, where Φ_TET-1_ (QD → BCA) and Φ_TET-2_ (BCA → annihilator) represent two sequential interfacial transfer steps. Although introduction of BCA increases the apparent interfacial extraction rate in TP-based systems (from 0.022 ns^−1^ to 0.035 ns^−1^), the overall Φ′_UC_ does not improve (6.9% for QD-BCA-TP vs. 7.9% for direct QD-TP), suggesting that parasitic losses during the relay process offset the faster initial extraction, as illustrated in Figure 4b,c. If we assume that in the QD-BCA-TP system all triplet energy transferred to TP originates from BCA, then the efficiency of Φ_TET-2_ can be estimated to be approximately 53.6%. This reasonable value helps explain why the overall upconversion efficiency does not improve despite the faster initial TET rate.

The direct sensitization observed in our ZnSe/ZnS-TP system aligns with recent reports demonstrating transmitter-free TET in ZnSe-based QDs. He et al. showed that trap-enabled long exciton lifetimes in ZnSe/ZnS QDs facilitate efficient triplet energy transfer to 2,5-diphenyloxazole without intermediate ligands, achieving visible-to-UV upconversion [46]. This mechanism likely contributes to the efficient direct sensitization observed here, as surface trap states can prolong exciton lifetimes and enhance the probability of diffusive encounters with annihilator molecules in solution.

The TP-versus-DTBN comparison establishes a clear hierarchy of design priorities in which annihilator fluorescence quantum yield outweighs the interfacial TET rate. Even with optimal interfacial engineering, a low Φ_PL_ annihilator fundamentally caps the achievable photon output. While prior studies have made significant progress in accelerating interfacial TET, our findings suggest that such efforts may be most effective when combined with the selection of annihilators possessing high fluorescence quantum yields.

## 4. Conclusions

In summary, we compared mediator-based (BCA) and direct sensitization strategies for visible-to-UV TTA-UC using ZnSe/ZnS QDs. Although the direct ZnSe/ZnS-TP composite exhibits a slower interfacial TET rate (0.022 ns^−1^) than its BCA-mediated counterpart (0.035 ns^−1^), it avoids cumulative energy losses associated with the transmitter ligand, resulting in a higher UC efficiency (7.9% vs. 6.9%). In contrast, DTBN shows faster TET in both direct (0.030 ns^−1^) and mediated (0.065 ns^−1^) architectures, yet its UC efficiency remains ≤1.5%, which can be partly attributed to its lower fluorescence quantum yield (4.77%). Thus, optimizing the energy transfer pathway, particularly minimizing interfacial losses, plays the primary role in achieving high UC efficiency.

## Figures and Tables

**Table 1:** Key performance parameters of the QD–molecule composites.

Sample	Φ_TET_ (%)	*k*_TET_ (ns^−1^)	Φ′_UC_ (%)
ZnSe/ZnS-BCA	22.5%	0.040	-
ZnSe/ZnS-BCA-TP	20.2%	0.035	6.90
ZnSe/ZnS-BCA-DTBN	32.1%	0.065	0.85
ZnSe/ZnS-TP	13.8%	0.022	7.90
ZnSe/ZnS-DTBN	17.9%	0.030	1.50

## Data Availability

The original contributions presented in this study are included in the article/Supplementary Materials. Further inquiries can be directed to the corresponding authors.

## Supplementary Materials

The following supporting information can be downloaded from https://www.mdpi.com/article/10.3390/nano16140843/s1. The supporting information includes (1) DFT-calculated singlet and triplet energy levels of DTBN, TP and BCA; (2) analysis of time-resolved photoluminescence kinetics of ZnSe/ZnS QD composites with TP or DTBN; (3) calculations of triplet energy transfer (TET) rates and efficiencies; and (4) determination of normalized upconversion quantum yields (Φ′_UC_); along with Figures S1–S11 and Tables S1–S3. References [14,15,45,47,48] are cited in the supplementary materials.
