# Supplementary material for: Transmitter-Free Interfacial Sensitization in ZnSe/ZnS Quantum Dots Enables Efficient Upconversion
Source: Nanomaterials (Basel). 2026 Jul 9;16(14):843. doi: 10.3390/nano16140843 (PMC13416163; doi:10.3390/nano16140843)
Supplement: Supplementary file 1 [file nanomaterials-16-00843-s001.zip › nanomaterials-4352531-supplementary.pdf]

Supporting Information

Transmitter-Free Interfacial Sensitization in ZnSe/ZnS Quantum Dots Enables Efficient Upconversion

1. Calculation of Energy Levels of DTBN and TP

2. Analysis of Time-Resolved Photoluminescence Kinetics

3. Calculation of Triplet Energy Transfer Kinetics

4. Determination of the Normalized Upconversion Quantum Yield

Figures S1-S11

Tables S1-S3

1. Calculation of Energy Levels of DTBN and TP

1.1 Experimental energy levels for the excited states S_1

The singlet energy levels (S_1) of the annihilators were estimated from the 0-0 transition energies (E_{0-0}), determined by the intersection of the area-normalized UV-vis absorption and photoluminescence spectra. The spectra were converted to an energy scale ($E \approx 1240/\lambda$) using the appropriate Jacobian transformation ($I_E = I_\lambda \cdot \lambda^2$) to ensure accuracy. The resulting intersection positions at 316.90 nm for DTBN and 310.31 nm for TP yield E_{0-0} values of 3.913 eV and 3.996 eV, respectively (Table S2).

1.2 Theoretical calculations for ground and excited states

The geometrical optimizations of stationary structures were performed using density functional theory (DFT) at the CAM-B3LYP/6-311G (2d, p) level as implemented in the Gaussian 16 software package. The lowest excited-state (S_1) geometries were optimized using time-dependent density functional theory (TD-DFT) calculations at the TD-CAM-B3LYP/6-311G (2d, p)//CAM-B3LYP/6-311G(2d,p) of theory [1]. The lowest triplet-state (T_1) geometries were optimized by unrestricted DFT at the UCAM-B3LYP/6-311G (2d, p) level of theory with the spin multiplicity set to 3. All optimized structures were confirmed as local minima by no imaginary frequencies on frequency analysis.

Table S1. Thermally corrected Gibbs free energies of the optimized S_0, T_1, and S_1 states of DTBN, TP and BCA calculated at 298 K, together with the corresponding adiabatic gaps relative to S_0.

Annihilator	S_0 / Hartree	T_1 / Hartree	Δ(T_1–S_0) / Hartree	Δ(T_1–S_0) /eV	S_1 / Hartree	Δ(S_1–S_0) / Hartree	Δ(S_1–S_0) /eV
**DTBN**	−699.834692	−699.74478	0.089912	2.446630138	−699.68493	0.14976	4.075177167
**TP**	−693.911134	−693.814367	0.096767	2.633164189	−693.76134	0.149799	4.076238411
**BCA**	−651.558861	−651.456429	0.102432	2.787316691	−651.40043	0.158427	4.31101825

Table S2. Photophysical Properties of DTBN and TP: Experimental and Computational Results.

Annihilator	Abs [a)] λ_{1st} (nm)	PL [a)] λ_{1st} (nm)	S_1 [b)] (eV)	T_1 [b)] (eV)	S_1 [c)] (eV)
**DTBN**	265	322	4.08	2.45	3.91
**TP**	270	323	4.08	2.63	4.00

a) In hexane; b) Determined from density functional theory calculations; c) Estimated from the absorption/PL spectra.

2. Analysis of Time-Resolved Photoluminescence Kinetics

To analyze the TRPL kinetics of ZnSe/ZnS QDs and their complexes, the decay traces were fitted with a bi-exponential function (Equation S1):

$$I(t) = y_0 + A_1 \exp\left(-\frac{t}{\tau_1}\right) + A_2 \exp\left(-\frac{t}{\tau_2}\right) \qquad (S1)$$

where I(t) is the PL intensity at delay time t and y_0 is a constant background. τ_1 and τ_2 are the lifetimes of the fast and slow components, respectively, while A_1 and A_2 are the corresponding amplitudes. The intensity-weighted average lifetime (τ_{avg}) was calculated using Equation S2:

$$\tau_{avg} = \frac{A_1\tau_1^2+A_2\tau_2^2}{A_1\tau_1+A_2\tau_2} \qquad (S2)$$

The fitting parameters and the calculated average lifetimes for all samples are summarized in Table S3.

Table S3. Bi-exponential fitting parameters and intensity-weighted average lifetimes (τ_{avg}) obtained from TRPL decays of ZnSe/ZnS QDs and the corresponding composites.

Sample	τ_{avg} (ns)	τ_1 (ns)	A_1	τ_2 (ns)	A_2	y_0	R^2
ZnSe/ZnS	7.25	1.48	17894	7.85	32201	210.5	0.9998
ZnSe/ZnS+TP	6.25	1.14	25464	7.10	24399	204.3	0.9997
ZnSe/ZnS+DTBN	5.95	1.30	29548	7.08	22343	182.2	0.9990
ZnSe/ZnS+BCA	5.62	1.65	21290	6.36	29577	167.4	0.9997
ZnSe/ZnS+BCA+TP	5.78	1.47	25667	6.69	26613	127.4	0.9992
ZnSe/ZnS+BCA+DTBN	4.92	1.17	31206	6.04	20176	168.1	0.9992

3. Calculation of Triplet Energy Transfer Kinetics

To quantify the triplet energy transfer (TET) dynamics from the ZnSe/ZnS QDs to the surface-anchored molecules, The TET efficiency (Φ_{TET}) and TET rate constant (k_{TET}) were determined from the intensity-weighted average lifetimes (τ_{avg}) extracted from TRPL decays (Section 3) using equations [2-3]:

$$\Phi_{TET} = 1 - \frac{\tau_{sample}}{\tau_{QD}} \qquad \text{(S3)}$$

$$k_{TET} = \frac{1}{\tau_{sample}} - \frac{1}{\tau_{QD}} \qquad \text{(S4)}$$

where τ_{QD} represents the intrinsic average lifetime of the pristine ZnSe/ZnS QDs (donor only), and τ_{sample} is the average lifetime of the QD-molecule complexes. The calculated kinetic parameters for all studied systems are summarized in Table 1.

4. Determination of the Normalized Upconversion Quantum Yield

TTA-UC measurements were carried out using a Spectra-Physics Spitfire Ti:sapphire femtosecond regenerative amplifier system. The 780 nm fundamental output was frequency-doubled in a β-barium borate (BBO) crystal to generate 390 nm excitation (1 kHz repetition rate, 120 fs pulse duration). The 390 nm beam was spectrally cleaned with optical filters to remove residual fundamental light and then focused into a 1 cm airtight cuvette (spot diameter ≈ 0.08 cm). The excitation power at the sample position was measured using a Thorlabs PM100D power meter equipped with a Thorlabs S401C thermal power sensor, and the corresponding power density was calculated by dividing the measured average optical power by the illuminated area. Thus, all reported power densities and threshold values correspond to average (not peak) power densities.

The upconverted emission was collected in the direction perpendicular to the excitation beam by directly positioning one end of an optical fiber near the cuvette wall, while the other end was connected to a fiber-coupled spectrometer (Ocean Optics) for spectral acquisition. The system response was calibrated prior to the measurements. Perylene in solution was used as the reference standard for photoluminescence quantum yield determination. The upconversion quantum yield (Φ'_{UC}) was calculated by a standard comparative method according to eq. (S5),

$$\Phi'_{UC} = 2\,\Phi_{Per}\frac{1-10^{-A_{Per}}}{1-10^{-A_S}}\frac{F_S}{F_{Per}}\left(\frac{n_S}{n_{Per}}\right)^2 \qquad \text{(S5)}$$

where A_i is the absorption (optical density) at the excitation wavelength (390 nm), F_i is the integrated photon number and n is the refractive index of the solvent. Where the quantum yield, absorbance, integrated emission intensity and refractive index of the

reference correspond to those of Perylene. Here, Φ'_{UC} denotes the normalized upconversion quantum yield, with the theoretical maximum defined as 100% [4-5].

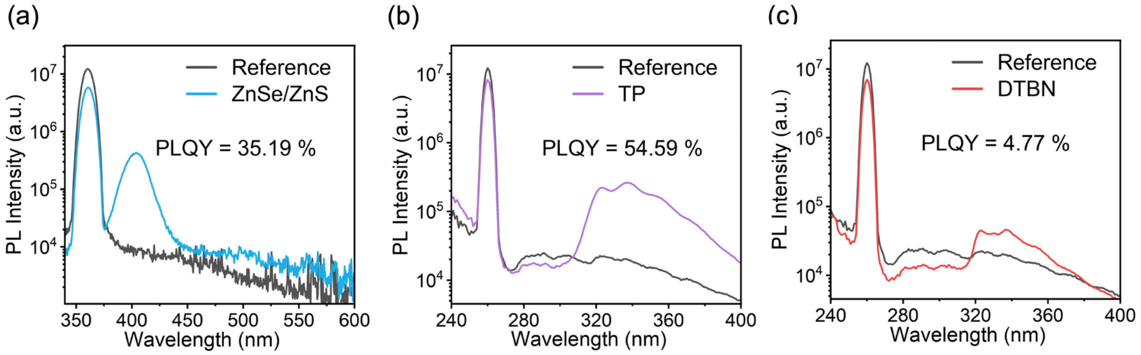

Figure S1. Steady-state PL spectra for PLQY measurements of (a) ZnSe/ZnS QDs, (b) TP, and (c) DTBN with their corresponding references. The excitation wavelengths were 375 nm for ZnSe/ZnS QDs and 260 nm for TP and DTBN. The PLQY values were calculated from the ratio of emitted photons to absorbed photons using an integrating sphere.

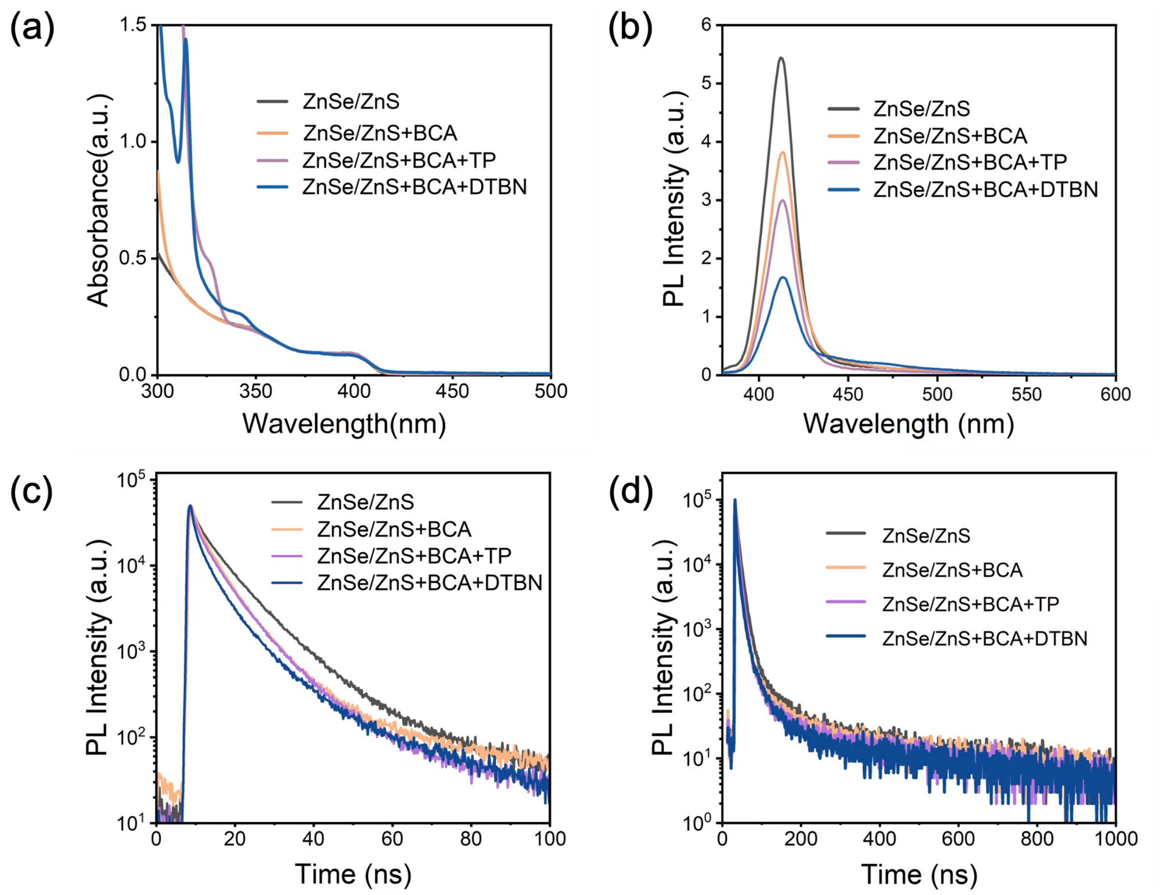

Figure S2. (a) UV-vis absorption spectra of ZnSe/ZnS QDs and the corresponding BCA- and annihilator-containing composites. (b) Steady-state PL spectra of the same samples. (c,d) Time-resolved PL decay traces plotted on a semi-log scale over (c) 0-100 ns and (d) 0-1000 ns for clarity. Samples: ZnSe/ZnS, ZnSe/ZnS-BCA (0.5 mM), ZnSe/ZnS-BCA (0.5 mM)-TP (3 mM), and ZnSe/ZnS-BCA (0.5 mM)-DTBN (3 mM). The steady-state PL spectra were measured under xenon-lamp excitation at 375 nm. The TRPL decays were measured under 375 nm laser excitation.

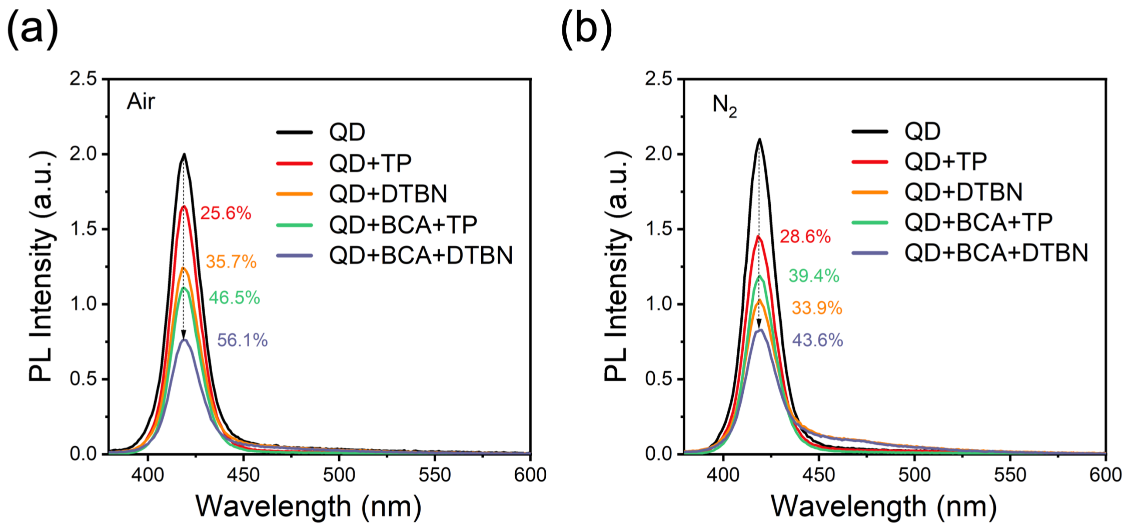

Figure S3. Steady-state PL spectra of ZnSe/ZnS QDs and QD–annihilator composite samples measured under 375 nm excitation in (a) ambient air and (b) N_2 atmosphere. The percentages indicate the PL quenching efficiencies calculated from the decrease in the integrated QD band-edge emission area compared with pristine ZnSe/ZnS QDs.

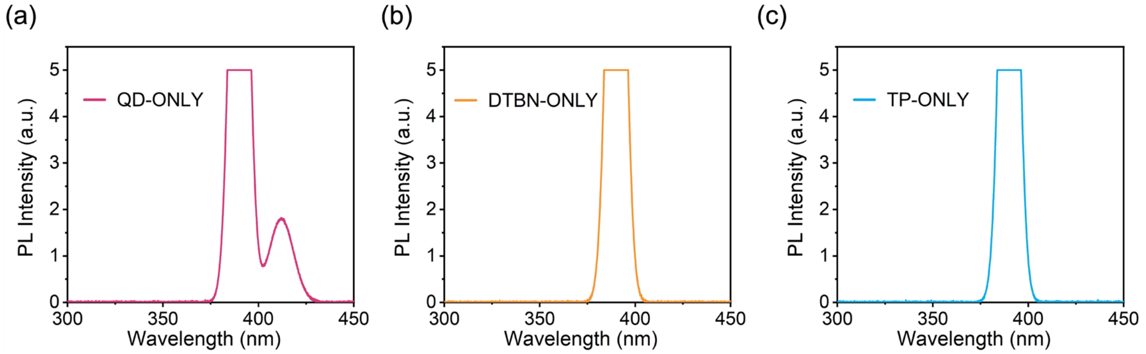

Figure S4. Control emission spectra recorded under identical excitation conditions: (a) ZnSe/ZnS QDs only, (b) DTBN only, and (c) TP only.

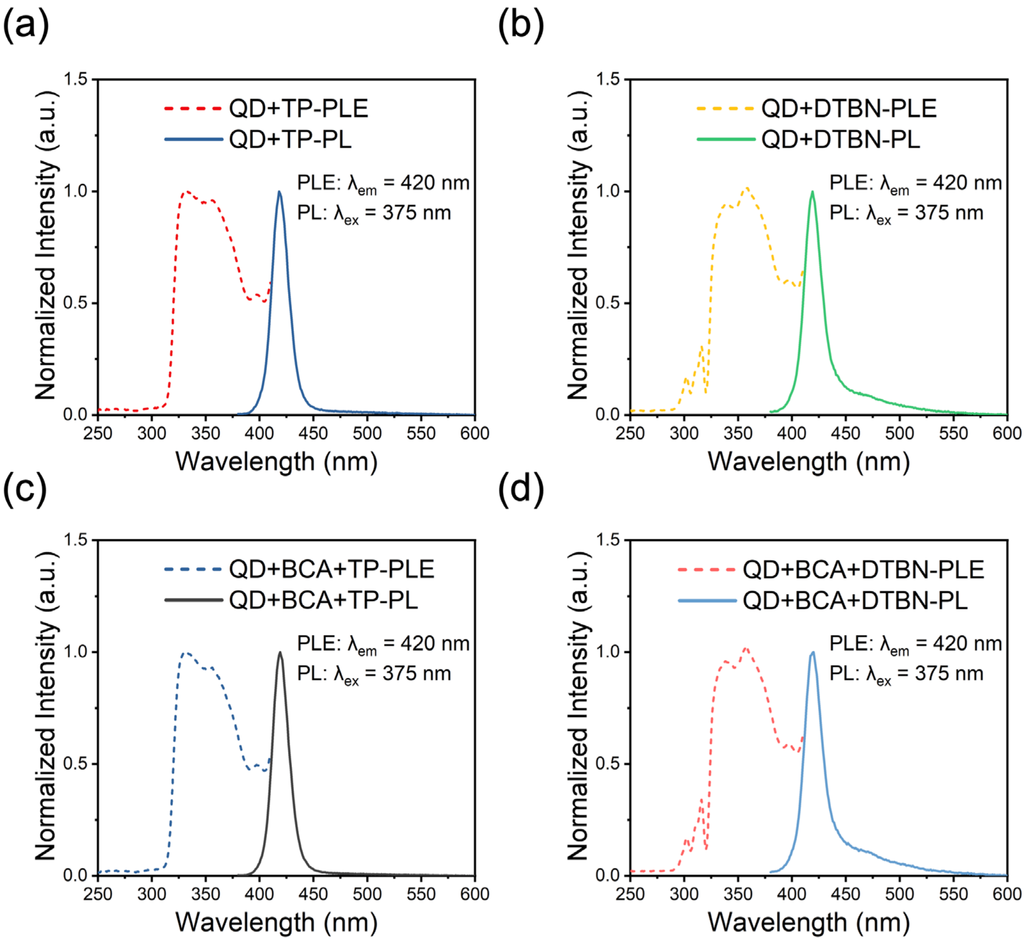

Figure S5. Normalized PLE and PL spectra of QD-annihilator composite systems: (a) QD-TP, (b) QD-DTBN, (c) QD-BCA-TP, and (d) QD-BCA-DTBN. The PLE spectra were recorded by monitoring the QD emission at 420 nm. The PL spectra were measured under 375 nm excitation. All spectra were normalized for comparison.

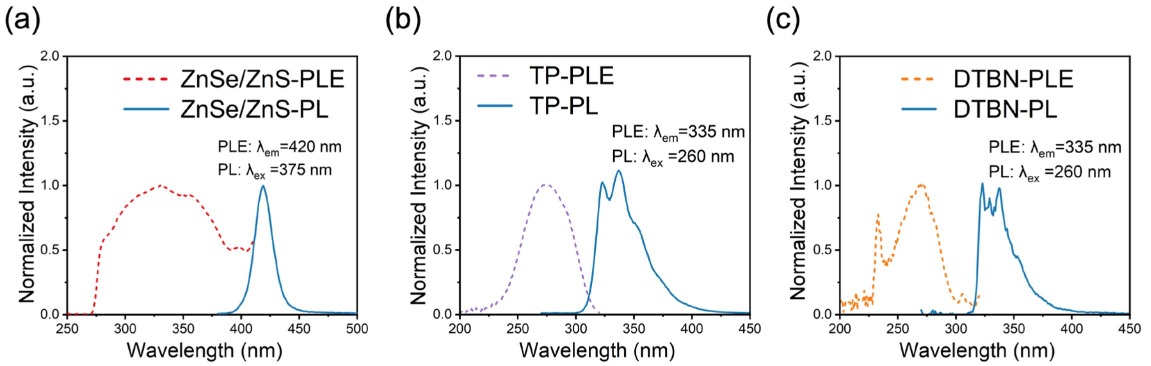

Figure S6. Normalized PLE and PL spectra of individual components: (a) ZnSe/ZnS QDs, (b) TP, and (c) DTBN. The PLE spectrum of ZnSe/ZnS QDs was collected by monitoring the emission at 420 nm. The PLE spectra of TP and DTBN were collected by monitoring the ultraviolet emission at 335 nm. The PL spectra were measured under 375 nm excitation for ZnSe/ZnS QDs and under 260 nm excitation for TP and DTBN. All spectra were normalized for comparison.

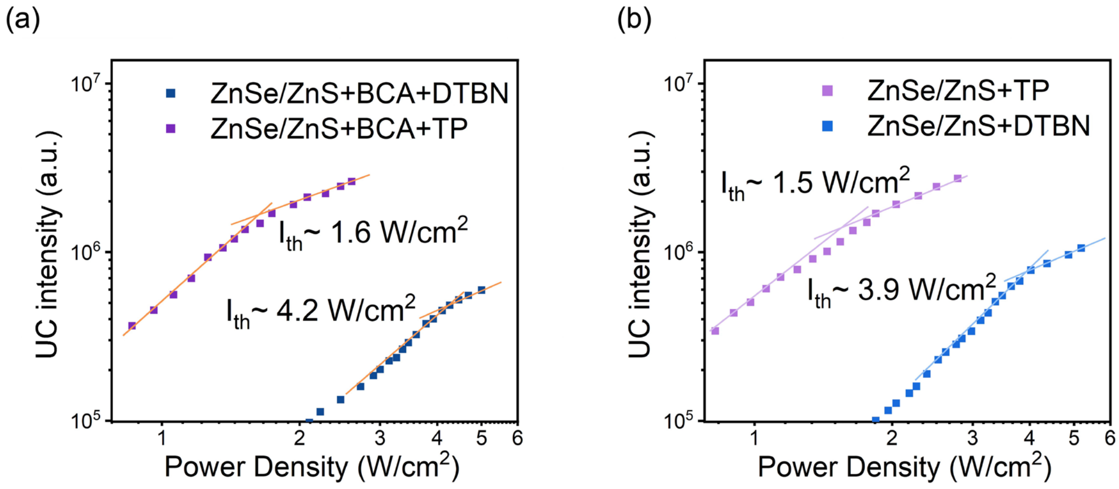

Figure S7. Log-log plots of integrated UC intensity versus excitation power density for (a) ZnSe/ZnS-BCA (0.5 mM)-TP (3 mM) and ZnSe/ZnS-BCA (0.5 mM)-DTBN (3 mM); (b) ZnSe/ZnS-TP (3 mM) and ZnSe/ZnS-DTBN (3 mM).

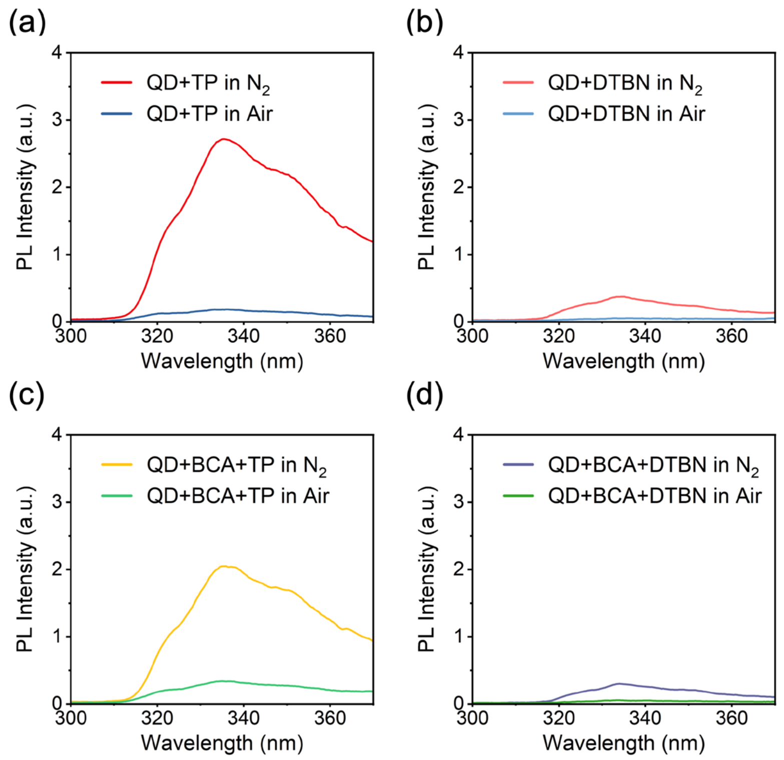

Figure S8. Upconverted emission spectra of (a) QD-TP, (b) QD-DTBN, (c) QD-BCA-TP, and (d) QD-BCA-DTBN measured under nitrogen and ambient air upon 390 nm excitation at an excitation power of 8.9 mW.

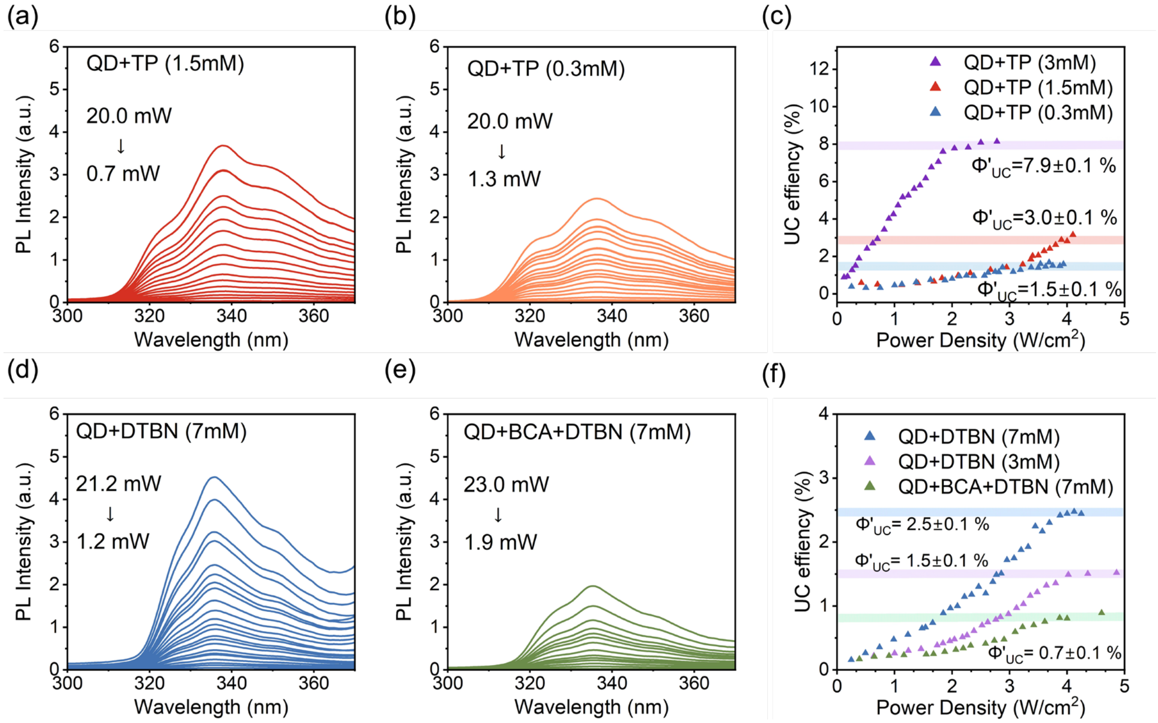

Figure S9. Power-dependent upconversion emission spectra of (a) QD-TP (1.5 mM), (b) QD-TP (0.3 mM), (d) QD-DTBN (7 mM), and (e) QD-BCA-DTBN (7 mM). Figure (c) and (f) show the corresponding normalized upconversion efficiencies versus excitation power density.

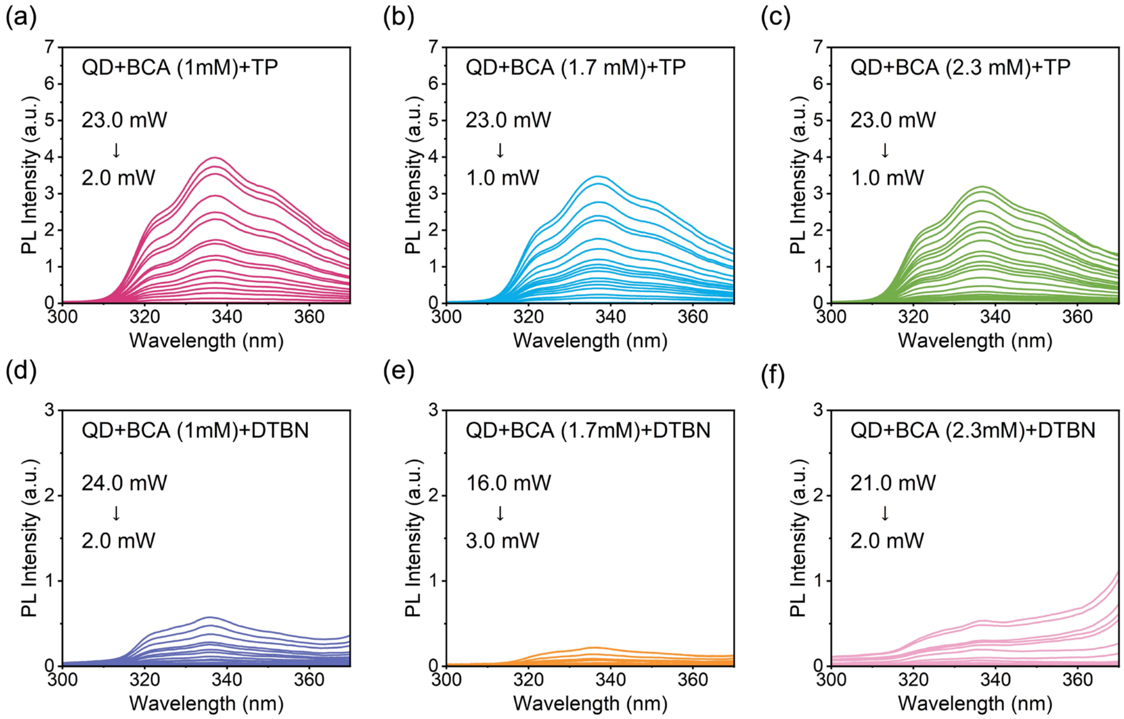

Figure S10. Power-dependent upconversion emission spectra of QD-BCA-annihilator systems with different BCA concentrations. (a-c) QD-BCA-TP with BCA concentrations of 1.0, 1.7, and 2.3 mM, respectively. (d-f) QD-BCA-DTBN with BCA concentrations of 1.0, 1.7, and 2.3 mM, respectively.

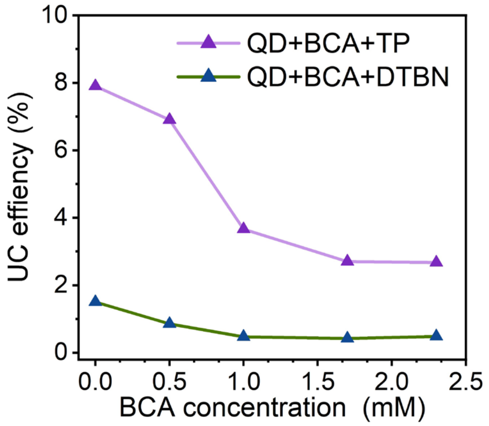

Figure S11. Upconversion efficiencies of QD-BCA-TP and QD-BCA-DTBN systems as a function of BCA concentration.

REFERENCE

[1] Yanai, T.; Tew, D. P.; Handy, N. C. A New Hybrid Exchange-Correlation Functional Using the Coulomb-Attenuating Method (CAM-B3LYP). Chem. Phys. Lett. **2004**, 393, 51-57.

[2] Mongin, C.; Garakyaraghi, S.; Razgoniaeva, N.; Zamkov, M.; Castellano, F. N. Direct Observation of Triplet Energy Transfer from Semiconductor Nanocrystals. Science **2016**, 351, 369-372.

[3] Luo, X.; Han, Y.; Chen, Z.; Li, Y.; Liang, G.; Liu, X.; Ding, T.; Nie, C.; Wang, M.; Castellano, F. N.; Wu, K. Mechanisms of Triplet Energy Transfer across the Inorganic Nanocrystal/Organic Molecule Interface. Nat. Commun. **2020**, 11, 28.

[4] Singh-Rachford, T. N.; Castellano, F. N. Photon Upconversion Based on Sensitized Triplet-Triplet Annihilation. Coord. Chem. Rev. **2010**, 254, 2560-2573.

[5] Zhou, Y.; Castellano, F. N.; Schmidt, T. W.; Hanson, K. On the Quantum Yield of Photon Upconversion via Triplet-Triplet Annihilation. ACS Energy Lett. **2020**, 5, 2322-2326.
